# Sarcopenia and Digitally Measured Free-Living Physical Activity in Older Adults

**DOI:** 10.1093/geroni/igaf122.1220

**Published:** 2025-12-31

**Authors:** Amal Wanigatunga, Lacey Etzkorn, Brian Buta, Sunan Gao, Josef Coresh, Jennifer Schrack

**Affiliations:** Johns Hopkins Bloomberg School of Public Health, Baltimore, Maryland, United States; Johns Hopkins Bloomberg School of Public Health, Baltimore, Maryland, United States; Johns Hopkins University, Baltimore, Maryland, United States; Johns Hopkins University, Baltimore, Maryland, United States; New York University, New York, New York, United States; Johns Hopkins Bloomberg School of Public Health, Baltimore, Maryland, United States

## Abstract

This study examined the association between sarcopenia (age-related muscle mass and strength loss) with digitally measured free-living physical activity patterns—measures that summarize biological, physiological, behavioral, and environmental effects on mobility. Among 853 participants in the Atherosclerosis Risk in Communities (ARIC) Study, sarcopenia at visit 9 was defined as slow gait speed (<0.8 m/s) and low hand grip strength (<20 kg/women, <35.5 kg/men). Accelerometry was collected using a 7-day, 24-hour wrist-worn protocol and summarized into activity amount (total activity counts/d), intensity (mean activity counts in the consecutive five most active minutes/d), endurance (activity bouts lasting ≥10 minutes/d), and accumulation (physical activity fragmentation %) patterning metrics. Linear regression was used to estimate cross-sectional associations between sarcopenia and each physical activity metric adjusted for age, sex, body mass index, gait speed, and grip strength. Mean sample age was 83 years, 58% were women, and 20% (n = 198) were classified as living with sarcopenia. After covariate adjustment, sarcopenia was associated with lower total activity (β=- 21,8158 counts/d, SE = 60,436; p < 0.001) and endurance (-24 minutes in activity bouts ≥10 minutes, SE = 10; p = 0.02). Conversely, sarcopenia was not associated with daily mean activity intensity (p = 0.13) or the way daily activity was accumulated throughout the day (p = 0.18). Results indicate that sarcopenia, above and beyond its individual components, is primarily associated with lower amounts of daily physical activity and endurance for movement related to everyday living. Our findings help define how sarcopenia connects to physical activity behavior in older adults and provides insight into potential preventative mechanisms.

